# Pride, Shame, and Group Identification

**DOI:** 10.3389/fpsyg.2016.00557

**Published:** 2016-04-26

**Authors:** Alessandro Salice, Alba Montes Sánchez

**Affiliations:** ^1^Department of Philosophy, University College CorkCork, Ireland; ^2^Center for Subjectivity Research, University of CopenhagenCopenhagen, Denmark

**Keywords:** shame, pride, group identification, self-conscious emotions, social self

## Abstract

Self-conscious emotions such as shame and pride are emotions that typically focus on the self of the person who feels them. In other words, the intentional object of these emotions is assumed to be the subject that experiences them. Many reasons speak in its favor and yet this account seems to leave a question open: how to cash out those cases in which one genuinely feels ashamed or proud of what someone *else* does? This paper contends that such cases do not necessarily challenge the idea that shame and pride are about the emoting subject. Rather, we claim that some of the most paradigmatic scenarios of shame and pride induced by others can be accommodated by taking seriously the consideration that, in such cases, the subject “group-identifies” with the other. This is the idea that, in feeling these forms of shame or pride, the subject is conceiving of herself as a member of the same group as the subject acting shamefully or in an admirable way. In other words, these peculiar emotive responses are elicited in the subject insofar as, and to the extent that, she is (or sees herself as being) a member of a group – the group to which those who act shamefully or admirably also belong. By looking into the way in which the notion of group identification can allow for an account of hetero-induced shame and pride, this paper attempts to achieve a sort of mutual enlightenment that brings to light not only an important and generally neglected form of self-conscious emotions, but also relevant features of group identification. In particular, it generates evidence for the idea that group identification is a psychological process that the subject does not have to carry out intentionally in the sense that it is not necessarily triggered by the subject’s conative states like desires or intentions.

## Introduction

The traditional account of shame and pride describes these emotions as self-conscious, i.e., as emotions that are intentionally directed at the very subject that feels them. But how can this account explain cases in which these phenomena, supposedly, are about others, i.e., cases in which the subject feels proud or ashamed of others?^1^ This paper has two main goals. The *first* is to show that the traditional account can successfully explain these cases if it is supplemented by a theory of group identification, where ‘group identification’ refers to the process that leads the emoting subject to understand herself as being member of a group (and, in the cases at stake, of the group to which the admirable or shameful others belong). The *second* goal is to employ such an account to shed light on group identification itself and, more specifically, to show that this is not an intentional process in the sense that it is not necessarily prompted and steered by conative states like the intention or the desire to belong to a group. For one can be trapped by one’s social identity, as it were.

To secure these insights, the paper proceeds as follows. In the *first* Section of the paper, the traditional account of shame and pride is introduced by focusing on the intentional structure of these emotions. In the *second* Section, the attention turns to pride induced by others – it is contended that this is an emotional response of a genuine kind and that, thus, it cannot be reduced to emotions with a different intentional structure or of an altogether different kind. One way to explain this form of pride, it is argued further, is by looking into the process of group identification – the subject, because she group-identifies, acquires a social self or identity, and it is such social self that her emotion of pride is about. In the *third* Section, this conclusion about hetero-induced pride is considered in the light of a psychological hypothesis according to which group identification is driven by a desire to achieve or maintain a positive social identity (cf. Tajfel and Turner, 1986). The following Sections peruse this hypothesis in the light of hetero-induced shame. If, as it is contended in Section 4, hetero-induced shame is an emotional response of a specific kind, then the conclusion we draw is that group identification is *not* necessarily triggered by a desire to attain or preserve a positive social identity: such a desire could well be a psychological factor in the case of hetero-induced pride, due to the fact that this is a positive emotion responding to what the subject perceives to be *positive traits* of a group to which she, then, wishes to be associated. However, as Section 5 shows, the fact that shame is an unpleasant emotion correlated to features of group identity perceived by the subject as negative, illustrates that such desire is not a necessary triggering factor of group identification. The paper ends by arguing that, if the desire to establish a positive social identity does not necessarily motivate group identification, then it is hard to see which other desires from the side of the subject might be able to motivate such a psychological process. If this consideration is on the right track, then it suggests that group identification is not necessarily an intentional process because it does not have to be triggered by conative states like intentions and desires.

## Shame and Pride as Self-Conscious Emotions

Despite the many rival views on the matter, in this paper we assign a central role in the characterization of emotions to appraisals or evaluations. This is in line with very widespread views in philosophy, and psychology too (cf. mainly appraisal theorists such as Scherer and Ekman, 1984; Smith and Ellsworth, 1985; Frijda, 1986, 2007; Lazarus, 1991; Scherer, 2009, 2013; Moors et al., 2013). Another widespread claim we also endorse in this paper is that emotions are intentional: insofar as these mental states are directed at objects and facts, they can be said to be *about* or *of* objects and facts (de Sousa, 2013). A peculiar class of emotions encompasses so-called “self-conscious emotions,” the peculiarity of which is that their intentional object coincides with the subject that feels them (cf. e.g., Kristjánsson, 2010, p. 77–85; Zahavi, 2012). Paradigmatic cases of self-conscious emotions are shame and pride: if I am ashamed or proud, I am ashamed or proud *of* myself. Some theorists also call shame and pride “emotions of self-assessment,” because they imply a self-evaluation by the emoting subject: whereas, in shame, I assess myself negatively, in pride, I assess myself positively (cf. Taylor, 1985; Tangney, 2005; Deonna et al., 2011). This is not supposed to insinuate that pride, shame and other self-conscious emotions are always products of self-reflection, of a mental process of rumination and almost solipsistic evaluation of oneself. Indeed, most instances of pride and shame are highly situational and they take the subject by surprise; this fact has even lead some authors to argue that these emotions may very well and often are pre-reflective (cf. Sartre, 1969). Indeed, some appraisal theorists argue that appraisal processes can often be automatic (cf. Anderson, 1992; Moors, 2010; Moors et al., 2013, p. 122). All that is meant here is that the situation that gives rise to self-conscious emotions is such that it urges the focus of the experience and the evaluation to turn onto the emoting subject. Accordingly, they differ from other emotions, like fear, whose focus and evaluation are, so to speak, “out there” *in* the world (if I’m afraid of the rabid dog, my experience focuses on it and evaluates it as threatening).

At this point, it might be important to highlight that all emotions are self-involving in some sense, they imply a relation between self and object, and often a triangular relation that includes also others (cf. Manstead and Fischer, 2001). This view is well articulated in appraisal theories of emotion, and many philosophers have developed theories that include similar components (cf. e.g., Helm, 2001; Roberts, 2003). Roughly, the idea is that all emotions involve the concerns of the emoting subject, they arise because the subject appraises the situation as having a significant impact on something she cares about, in the sense that the situation affects her. Thus fear does involve a concern for oneself. And yet, it doesn’t involve a self-evaluation. In fear, a situation is appraised as threatening to me or someone or something I care about. In pride and shame, by contrast, the situation is experienced as revealing that *I* am, respectively, superior or inferior, outstanding or degraded… and these are all evaluations *of myself*.

All of the above makes the intentional structure of shame and pride virtually symmetrical: if, on the one hand, their phenomenological qualities are basically opposite (one being positive, the other negative), the way in which they relate to facts and objects, on the other hand, is almost parallel.^2^ Two considerations can further illustrate the symmetry between these emotions. *First*, both emotions entail the sense that one is in the spotlight, that something about oneself is being revealed and brought to the fore (Kristjánsson, 2010, p. 77). However, whilst in pride one is typically satisfied about it and shows it off, in shame one wishes and seeks to hide it again. In pride, one feels an expansion of the self, a swelling, and a desire to share the good news or show to others the good qualities that make the subject proud (although this reaction can be constrained by culture). In shame, exactly the opposite happens: the body adopts a collapsed position, one hides one’s face and wishes to run away or sink through the ground and disappear from the view of others.

Granted, this characterization does not account for the complexity of pride and shame, which can come in much more intricate forms. Sometimes pride can be uncomfortable or mixed with embarrassment, especially if one feels one is being excessively praised, and sometimes shame can be mixed with pleasure, as might be the case in the first sexual encounters of a young person. Such complexity, however, only strengthens the hypothesis of a structural similarity between them. It does so by pointing toward the idea that these emotions build upon the same ground: if considered from a developmental perspective, self-conscious affectivity appears to be rather ambivalent – the first coy reactions of babies seem to indicate that their feelings are neither entirely pleasant nor entirely unpleasant (cf. Reddy, 2000). Later on the reactions and experiences appear to differentiate and become hedonically “purer” (Reddy, 2008, p. 120–149), although many stay mixed even in adulthood.

The suggestion of an intrinsic similarity between shame and pride can be supported by a *second* consideration: the intentional structure displayed by these emotions is peculiar and makes them different from simpler emotions like, e.g., fear. The latter shows a relatively simple structure: if a child is afraid of darkness, then her fear is intentionally directed at darkness *as* something threatening. The expression “as something threatening”^3^ is meant to capture the idea, widespread in the literature, that emotions enter relations with so called “formal objects” (cf. Kenny, 2003, p. 134), which provide adequacy criteria for emotions: accordingly, fear is adequate if it responds to an object which is threatening; anger if relates to the object as offensive, and so on.

Self-conscious emotions, by contrast, are more complex insofar as they involve a subdivision of their intentional objects into the object proper and what one may call the cause of the emotion (Hume, 1978; Taylor, 1985). Imagine that you are a scientist that, after years of efforts and hard work, wins the Nobel Prize for Physics, and you feel proud of it. In this case, the best way of capturing the intentional structure of your emotion is not by saying simply that you are proud *of the prize*. Your pride does not *merely* focus on the prize in the same way in which fear focuses on threatening objects, it rather focuses on the *winning* of the prize, which is something *you did*, and which directly reflects upon you. It is plausible to contend that, without this connection, you wouldn’t feel pride. Such a structure is essential to these emotions: you feel proud *of* yourself *because* you won the prize. You are the object of your pride, and the cause or occasion makes you evaluate yourself positively (cf. Hume, 1978; Taylor, 1985). Shame has the same structure. Imagine a teenager who was ashamed of his acne. His shame doesn’t focus on the features of a young face covered in pimples (indeed, he might even be inclined to make fun of them if he saw them on someone else); it focuses on the fact that it is *his* face, and this leads to a negative evaluation of himself. He is ashamed *of* himself *because* his face is unattractively covered in pimples.

In his *Treatise of Human Nature*, Hume (1978, p. 277–290) analyzes both pride and shame along the lines sketched above.^4^ According to him, both have a primary intentional object (the emoting subject) and a cause. But for the cause to give rise to a self-conscious emotion (like pride or shame), and not to another type of emotion (admiration or disgust), the cause must be, as he says, “closely related” to the emoting subject, or, one could add, it must at least be experienced as such (Hume, 1978, p. 291). Many different things can serve here as a cause: a feature, an action, an object, a situation, as long as it bears this close relation to the emoting subject. The crucial point is not what type of thing or event it is, but such close relation to the subject. This relation implies something more than the mere subject-object relation, which the subject’s concerns make intrinsic to basically all emotions. When Hume refers to the close relation between subject and cause in this context, he doesn’t merely refer to an impact on concerns. He rather means that there is a link of some kind that allows for the evaluation deserved by the cause to be transferred to the emoting subject: if the cause produces pleasant impressions in me, I will feel good about myself, and therefore feel proud, and if these impressions are unpleasant, I will feel bad about myself, and therefore feel shame. According to Taylor (1985, p. 28–32), in this context the adverb “closely” for Hume means that there must be something about this object or situation that can have an impact on one’s sense of self. There has to be what she calls a relationship of belonging, a relationship that allows for identification: for whatever reason, the subject has to perceive that her or his identity is at stake or affected in this situation. Pride- and shame-inducing objects and situations will vary depending on culture, character, personal values and so on, but they will share this capacity of impacting one’s sense of self.

This idea of relation, however, seems to be in need of further exploration. On the one hand, the way in which your achievements, for example, reflect on yourself and allow for self-assessment can be seen as rather unproblematic: they reveal some positive qualities of yours, like your hard-working determination, or your talent, or what have you. Even Hume’s example of the man who is proud of his house (Hume, 1978, p. 310–311) can be construed along these lines: his choice of house might be seen as revealing the man’s exquisite taste or his good social position, and thus granting a positive self-assessment. But, on the other hand, what about feeling proud of other people’s qualities, actions or achievements? Do these not rather reflect on these other people? Why should they reflect on oneself instead and cause pride? And yet, this happens all the time. If your daughter won the Nobel Prize instead of you (and you got to see it), you would probably feel equally proud, if not even prouder, than if you won it yourself. Indeed, many parents often feel proud of their children for everyday achievements, and it is not rare for many of us to feel proud of others: of our close friends or family members, of members of our community that do admirable things, of the sports team we support, and so on.

If it is true that we can feel proud or ashamed for actions that *others* have performed (or sometimes even qualities they have), how are these cases to be squared with the standard account? Or does this mean the standard account has to be dropped and a new one that does justice to this fact is to be devised (cf. Helm, 2010, p. 106)? We do not think so: the standard characterization of shame and pride as self-conscious picks out a distinctive phenomenological feature of these experiences, and is supported by developmental (Reddy, 2008) and evolutionary (Maibom, 2010) evidence. Shame and pride, as opposed to other emotions that involve assessments of others (like disdain, indignation, or admiration), are both characterized by a specific form of self-experience: they throw us back upon ourselves in a way other emotions don’t do, they make us feel exposed (cf. Sartre, 1969, p. 252–303; Zahavi, 2012). Developmentally, they start as reactions to experiencing other people’s attention to oneself (Reddy, 2000, 2008), and evolutionarily they seem to descend from mechanisms to signal that an individual is assuming a specific status vis-à-vis another as a way of regulating conflict (Maibom, 2010). Motivationally, they imply opposite tendencies to hide ourselves (shame) or to show off (pride), and these do not change significantly when we are ashamed or proud of someone else. A typical reaction to feeling ashamed of someone would be to retreat from the situation or make oneself as invisible as possible, and alternatively to hide any link to the shameful subject. As for taking pride in others, most people have encountered proud parents and grandparents who can’t help boasting about their children. This boasting is in most cases significantly different from praising someone one admires: compare the case of a proud parent with the attitude one might take in recommending to one’s friends the concert of a talented musician one just discovered. Pride and shame of others still is phenomenologically self-directed in some sense. The aim of the following Section, therefore, is to clarify in which sense this is the case and to defend the standard account.

At this point, one might object we are forgetting an important ingredient: social appraisals, i.e., the important impact that the experienced or anticipated reactions of others (their thoughts, emotions, and actions, etc.) to the situation at stake and our emotions about it can have on the way we experience that situation, and thus shape our very emotions (Manstead and Fischer, 2001). Social appraisals often feature in our emotions and alter them significantly, and they may perhaps explain how we can come to feel ashamed or proud of others. Now, it is undeniable that social appraisals play a role, but they do so too for standard cases of shame and pride: we don’t see a significant difference here. In mature adults, there can perhaps be cases of solitary shame or pride, where all that is at stake is the individual’s own independent self-evaluation, but other people’s attention to and evaluations of oneself feature centrally in most instances of these emotions, they are essential for their developmental genesis, and they greatly increase their felt intensity. Granted: our sense of self is less individualistic than some approaches to emotion seem to assume, i.e., it is essentially relational (Manstead and Fischer, 2001, p. 224). And many situations can make us feel associated with others through social appraisals. But what does this association consist in? The aim of this paper is precisely to clarify how exactly this relationality gets expressed in hetero-induced shame and pride.

Let us now take a step back to the idea that shame and pride are self-conscious emotions. There are at least two ways in which the claim about the self-reflectivity of these emotions can be defended in the light of their hetero-induced forms. The *first* is to recur to a reductivist strategy and to argue that there is nothing genuinely specific about the hetero-induced emotions depicted in the examples above. Section 2 begins by applying this strategy to pride induced by others’ actions and rejects its conclusions. But then, if these emotive responses are to be vindicated as genuine instances of pride, how is one to explain them? The *second* way to preserve the standard account, and the one we endorse in this paper, is by supplementing it with a theory of group identification; to feel what in the following will be called ‘hetero-induced’^5^ shame and pride, the subject has to group-identify with the individual acting admirably or shamefully. It is *because* the subject understands herself as belonging to the same group to which the admirable or shameful agent belongs that she feels pride or shame – accordingly, these emotions would still be about the self, but the self at stake is the *social* self of the experiencing subject. Put differently, this form of pride is pride of oneself *qua member of a group*, the group to which the shame- or pride-inducing agent is perceived to also belong. As (perceived) members of the same group, the emoting subject and the agent that causes the emotion would share a social identity, which enables the subject to feel that self-conscious emotion.

A linguistic remark before addressing all this in more detail. Although we do not consider this paper as a contribution to ordinary language philosophy, we would like to point out that some natural languages have expressions that, to some extent, track the specificity of the emotions we are after. This is especially the case for hetero-induced shame, which to some extent can be expressed by the German singular term ‘Fremdscham’ and by the Spanish ‘vergüenza ajena.’ Also, the Italian language allows the compound expression ‘vergognar*si* di qualcuno,’ which is reflexive. The language of pride is not equally rich,^6^ but some romance languages, like Spanish, do use reflexive structures to refer to hetero-induced pride: ‘enorgullecer*se* de alguien.’ English does not allow for this, however, and the English expressions ‘being proud *of* someone’ and ‘being ashamed *of* someone,’ which will be used in the following, might be taken as misleadingly suggesting that the intentional object of these emotions is someone else (instead of its very subject). Interestingly, the English use of the expression ‘being ashamed,’ which can be said either of the emoting subject or of the one inducing shame, reflects the way in which the Japanese term 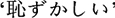 (hazukashii) is used – as both can be applied either to the subject feeling shame or to the individual that causes shame in an experiencing subject. But this is just to show that, although ordinary language may be granted the first word in the description of emotions, it seldom has the last. Put another way, the aim of this paper is not to describe how the best speakers of one or the other language use these emotion terms, but rather to understand the structure of these experiences. Hence, it is now high time to turn to the phenomena themselves.

## Being Proud of Your Daughter

Back to the example: Your daughter wins the Nobel Prize (or, perhaps, much more ordinarily, and yet not less moving, she takes her first steps) – and you feel an emotion that, from all angles, feels and looks very much like pride. But then you might wonder in which way *you* have contributed to that event to the effect that you, *yourself*, now feel proud because of it. In other words, the following problem arises: given that pride is a self-conscious emotion (directed back toward the emoting self), then it is not clear how it comes about that you, yourself, feel proud – of yourself! – in the light of an event to which you have not contributed in any way. A first way to tackle the problem is by denying that here one is confronted with a case of pride, or by arguing that, on close examination, such cases do not really differ in any way from standard non-puzzling examples of pride. In particular, it could be argued (a) that this emotive response is an emotion of pride *tout court* to be aligned to standard cases of pride; or maybe (b) that this is a somewhat mystified emotion of admiration (i.e., an emotion which is a cognate with – and yet different in kind from – pride); or (c) that this is an emotion of pride that has not been elicited in the ‘standard’ way, but rather by a process of emotional contagion; or (d) that this scenario, again, involves an emotion of pride – but that this is a “fictional” form of pride. In this paper we scrutinize these four attempts, reject all of them and develop an alternative account – one that retains the phenomenological credentials of this emotion and yet accommodates its hetero-induced form by enriching the standard account with a theory of group identification. Let us remark at this stage that the claim is *not* that the aforementioned options are unable to model certain forms of pride – quite the contrary: they capture phenomena that are real and perhaps even frequent. Rather, the claim is that none of these options can account satisfactorily for the most paradigmatic examples of feeling proud of someone else and, hence, that they leave this phenomenon unexplained.

We begin by excluding the first reductivist move as a blunt non-starter. If presented with this example, one might argue that, since you are her parent, your daughter’s achievement reveals to you *your* good parenting skills (in the same way that your choice of house may reveal your excellent taste, as in Hume’s example). However, this interpretation just doesn’t do justice to this experience. To claim that, in feeling proud of your daughter, you just focus on yourself and your parenting skills, simply fails to capture the crucial sense in which her and her achievement feature as central in your experience.

But then, could hetero-induced pride simply be a form of admiration? If, in the example at stake, there is an important sense in which you are proud *of your daughter* and you are focusing on her and her achievement, then one could perhaps claim that, the target being another person, there is nothing here but admiration (and not pride), where ‘admiration’ is understood as a positive emotion in response to, in this case, an outstanding person (cf. Schindler, 2014). And yet, it seems that the feeling at issue is fairly different from mere admiration. You might very much admire the previous Nobel Prize winners, but you are not likely to feel proud of them (although you might, under some conditions we specify below). Feeling proud of someone else, as opposed to admiring them, preserves the phenomenal qualities of self-expansion and self-assessment: the winner is not just any excellent scientist, it’s *your* daughter. Something good about yourself is after all revealed. It seems clear that, derivatively, and without losing sight of the fact that it’s her achievement, not yours, you feel good about yourself, and probably even inclined to boast about it.

Are we then facing a ‘standard’ case of pride that, however, has been caused in a somewhat peculiar way? Put differently, even if one grants that the emotion at stake in the example is of the kind of pride, one could still try to water-down its peculiarity by arguing that, in these cases, we just face an emotion triggered by emotional contagion. Emotions can be “infectious” (cf. Goldie, 2000, p. 189), they can pass from one individual to another by means of contagion. Think, e.g., of how your mood can easily be contaminated by the jolly atmosphere of a bar, once you enter it. Similarly, one could claim that hetero-induced pride is nothing else than individual pride that has passed from one individual to one another. This thought might be promoted by the frequent use of the adjective “vicarious” to designate hetero-induced emotions (cf. Lickel et al., 2005; Welten et al., 2012; Yamawaki et al., 2015). But as Scheler (1987) rightly pointed out, there is a crucial difference between what he considers properly vicarious emotions, i.e., emotions caused by contagion, and genuine forms of hetero-induced emotions. Consider how the explanation in terms of emotional contagion falls too short of our example of feeling proud of your daughter: first, you don’t need to witness anybody else feeling proud in order to feel that emotion yourself. Imagine, for example, that before anyone had a chance to break the news to you, you read a newspaper headline announcing your daughter as the winner of that year’s Nobel Prize. Reading this headline, just learning that she was awarded the prize, is very likely to make you feel proud, with no need for you to witness or imagine her feeling proud. What you are feeling is your own emotion, not something you picked up from somebody else. In addition, and most crucially, it could even be that your daughter is *not* feeling pride (maybe because she has a very humble character), but you do, and this would invalidate the appeal to emotional contagion in the very first place.

Another possibility to maintain the idea that the emotive response at stake is pride, while yet denying that there is anything specific about it, is to argue that these are cases of “fictional” pride, i.e., cases of an emotion that one can come to feel by putting oneself in somebody else’s shoes. This is something that happens often when we enjoy works of fiction and identify with a character: sometimes we come to feel what the character feels, while keeping a sense that we are not really that person and that the states of affairs represented in the fiction do not obtain. This is why Walton (1978) famously (and controversially) argued that what we experience here are “quasi-emotions.” Mechanisms like this might also be at play, for example, when we see someone do something dangerous and we feel afraid. But does this really explain hetero-induced pride?

To begin with, there is a *prima facie* difference between fictional and hetero-induced emotions: fictional emotions seem to require that you put yourself in *somebody else’s* situation, while hetero-induced emotions arguably do not require this, for they arise out of your own situation.^7^ To see the difference this makes, consider these following scenarios. While watching Ridley Scott’s *Alien* in the cinema, you may feel very intensely a range of fictional emotions, including fear. This is partly because the way in which the fiction is constructed leads you to put yourself in the characters’ position and feel the emotions appropriate to their situation. But you are very unlikely to *feel* afraid if someone simply told you the film is about a crew of astronauts trapped in a spacecraft with a murderous alien creature that hunts them: you need more than this to put yourself in the position of the astronauts. By contrast, you don’t need to put yourself in your daughter’s shoes to feel proud of her. When you read the newspaper headline announcing her prize, there is no need for you to adopt her perspective toward that event, so that you can feel the pride that would be adequate to her own situation. Put another way, you don’t need to simulate her pride in yourself. This emotion is your own: it arises from *your* situation. This signals an important difference between feeling the pride that your daughter feels, or would potentially feel (in which case the emotion would be purely “fictional” in the above sense), and feeling proud *of* your daughter, which is *your* feeling (and hence, a hetero-induced emotion in our sense).

If these considerations are correct, they show that hetero-induced pride is an emotive response with a specific phenomenology. Again, the hypotheses reviewed and rejected above describe phenomena, like fictional emotions, that we believe are possible, perhaps frequent. They may be routes through which some actions and features of others can have an impact on our self-conscious emotions. But they fall short of explaining some of the most paradigmatic examples of pride induced by others, which qualifies as a genuinely specific emotive response. But this opens the question of how, then, one is to explain this emotion.

As already suggested, one way to look at hetero-induced emotions is to argue that the subject, to feel this emotion, needs to group-identify. But what does that exactly mean? To illustrate this idea, it might be important to appeal to an established paradigm in social psychology, generally labeled “Social Identity Theory,” according to which individuals do not only have a personal identity, they also have one or more social identities or social selves. One’s social identity does not have to be conceived as something different from one’s personal identity – rather, one’s social identity could be said to be a part of one’s personal identity. The social self can be intended as the representation one has of oneself *qua* member of a *group.* One straightforward way to look at it is by connecting it to the process of self-categorization (Brewer and Gardner, 1996).

Self-categorization seems to be elicited by the individuals starting to perceive of themselves as being *similar* to others *in a certain respect* or as sharing certain properties with others. The properties can be of a manifold nature: social properties such as “being an African American,” “being a phenomenologist,” “being an Anglican,” “being an anarcho-syndicalist” can be used to characterize oneself, e.g., within an academic, political or a religious context. But empirical evidence (Tajfel, 1970; Tajfel et al., 1971) seems to show that even much more minimal properties (in the sense of properties negligible in usual contexts) like “preferring Beethoven to Mozart” or “preferring Paul Klee to Wassilj Kandinski” could acquire salience and be put at the basis of self-categorization. When this occurs, so-called ‘minimal groups’ arise – these are groups that have a purely cognitive existence in the sense that they exist only in the mind of the individuals, as it were.

What seems to be important for the purposes of this paper, however, is that the mere understanding of belonging to a class or group, i.e., to the group identified by a given property (e.g., the class of analytic philosophers, the class of the Beethoven enthusiasts, etc.) is *not* yet *per se* to identify with that group (Bennett and Sani, 2008). The subject, who is aware of exemplifying certain properties, does not yet have to articulate her experiences by using the first person plural pronoun or, said another way, to frame the situation she is in according to a we-perspective. In a sense, the subject does not yet live through the group from within and the mere idea of belonging to a specific class of individuals does not necessarily affect the way in which one behaves, feels or thinks. But this is exactly what it means to see oneself as a member of a *we* or, even, to adopt a we-perspective, which appears to imply a peculiar switch from a mere spectator to a participatory perspective. Indeed, a wealth of psychological literature identifies marked predispositions to altruism toward in-group members, to emotional sharing, to sympathy, to collective actions, to *we*-talk, etc. (Turner, 1987, p. 50) as quintessential byproducts of group identification.

If group identification can be triggered by, but does not coincide with, self-categorization, then it might well be the case that self-categorization is just one among many routes that lead the subject to adopt the we-perspective. Indeed, in certain cases, dyadic forms of intentionality (as instantiated in face-to-face communication, e.g.) can lead the individuals involved conceiving of themselves as *us* (cf. Zahavi, 2015). And perhaps other processes or situations as well could be argued to be conducive to group identification (Bacharach, 2006, p. 76). We will come back to this notion in the following Sections, but for the present purposes, suffice it to say that, once the individual has group identified, the subject’s self-consciousness undergoes a specific transformation: the *self* is now sublated under a *we*, a *we* to which also others are sublated and to which the self feels attached. In other words, the individual has acquired a *social self*, i.e., she now has a representation of herself qua member of us.

Against this background, we are now in a position to provide an account of hetero-induced pride, which converges in some important respects with some psychological theories about intergroup, or group-based, emotions (Smith et al., 2007; Ray et al., 2014).^8^ When you feel proud of your daughter, the emotion is still about yourself, your self, but this is about your self insofar as it is *your social self*. Seeing yourself as a member of a group, the actions and/or achievements of the other members acquire relevance when it comes to assessing your *social* self, and this is what triggers the emotive response (cf. Lickel et al., 2007). Accordingly, it is precisely because the Nobel Prize has been won by a close member of your group – namely of your family – that such an achievement has relevance for you and for the way in which you assess yourself.^9^

At this stage, note that the emphasis on the self-conscious nature of hetero-induced emotions does not amount to disregarding the role that the other (your daughter, in the example at stake) plays within the intentional structure of the emotion. After all, if this emotion is *induced* by others, there must be an intentional relation that the emotion is required to enter with such others. Yet, it seems plausible to contend that this relation is different from the one entered by the self of the emoting subject. The latter fills the emotion’s *target* position (just as, in fearing the dog, the dog is in the target position): it is your self (as group member) of which you are proud. By contrast, the former, the other person, is in what has been called the “focus’s position” of the emotion – where the focus can be characterized as a “background object having import to which the target is related in such a way as to make intelligible the target’s having the property defined by the formal object” (Helm, 2010, p. 58). Put differently, it is in virtue of the focus of the emotion (i.e., the other and her actions) that the target of the emotion (i.e., the social self) acquires the property defined by the formal object (for instance, being admirable).

Putting the social self in the target position of the emotion might allow explaining a certain variance in the phenomenology of these affects. In certain cases, the self-referentiality of these emotions is immediately reflected in the way in which the emotion is felt by the subject – e.g., consider cases in which a fan feels proud of him- or herself because his or her sport team has won the match: the fan generally attempts to underline the fact that he or she shares the same social identity with the team (this example will be discussed further in Section 3). In others, by contrast, it is the other who appears to play a more ostensive role. To see this, consider how parents feeling pride of their children, while talking to other parents, direct the hearers’ attention to their children rather than to themselves (and yet these still are *their* children).

Interestingly, the phenomenological difference between these cases manifests itself in degrees and does not impose itself as clear-cut. It is difficult to pinpoint when, exactly, the other’s position in the emotion turns from an implicit to an explicit or ostensive (and vice versa). This seems to suggest that an explanation of this oscillation between implicitness and explicitness should be given in terms of emphasis rather than in terms of two intentional structures that are instantiated by two different kinds of hetero-induced affects. More precisely, we propose that, in some cases, the emotion of pride (or shame) is “more” centered on the subject, while, in others, it is “more” centered on the other. But this oscillation occurs – and is grounded in – the social self that is and remains the proper target of the emotion: in the first scenario, the phenomenological accent is put on *me* being the member of the group to which the other belongs. By contrast, in the second case, the phenomenological accent is put on the *other* being the member of the group to which *I* belong. If this correct, then these two allegedly different targets of the emotion just are two poles of one and the same target (the social self).

## Group Identification and its Underlying Motivation

If this line of thought is so far correct, there appears to be a form of pride, i.e., hetero-induced pride, that is based on group identification. Hence, complementing the theory of self-conscious emotions with a theory of group identification seems to be a germane move insofar as it helps shed light on a specific form of such emotions. But is there any insight about group identification that one could gain by considering this notion in the light of the self-conscious emotions that this process can contribute to trigger?

Before tackling this question, it might be helpful to emphasize a point made above: “objective” group membership, i.e., the fact that one objectively belongs to a class or group defined by a socially salient property, does not automatically lead to subjective identification with that group. One can be perfectly aware of working for a given company, for example, and not feel any particular inclination to identify with it or frame its actions or interventions in the public sphere in we-terms. Put another way, one can be a member of a group without forming a (corresponding) social self. Conversely, in some situations it is possible for people to identify with groups to which in principle they do not belong, or that do not exist in any robust sense, or whose borders and defining properties seem so blurry as to throw serious doubt on whether one can call them groups or not (think again of the abovementioned “minimal group” scenarios). It seems therefore clear that group identification does not always neatly align with group membership. If this is the case, then what leads people to group-identify?

The foregoing analysis of hetero-induced pride could tempt one to think that group identification is triggered by a desire to share a positive social identity – since the subject intends to share a social identity that is perceived as positive and admirable, she begins to understand herself as a group member or, in other words, she group-identifies with another individual. Some researchers have argued that, at least in paradigmatic cases, a crucial assumption to be made in order to explain group identification is that “individuals strive to maintain or enhance their self-esteem: they strive for a positive self-concept” and that this principle sustains the idea that “individuals strive to achieve or maintain positive social identity” (Tajfel and Turner, 1986, p. 16). If considered under the light of hetero-induced pride, this assumption has high plausibility. Indeed, it might seem tempting to conclude that group identification is *always* motivated by a desire to belong, which aims at assimilating the positive qualities of others and attributing them to oneself (cf. also Brewer and Gardner, 1996). Roughly, the idea would be that group identification is the result of some sort of practical reasoning, where one of the premises is the desire of being associated with a positive group’s identity. The scheme of such reasoning could run along the following lines:

(i) The subject believes that a given individual (or a given group, for that matter) is associated with positive qualities,
(ii) The subject desires to be associated with those qualities, hence,
(iii) The subject group-identifies, i.e., she starts to think of herself as member of the same group to which the other individual belongs (or to the group *tout court*).

Once the condition pointed by (iii) is achieved, the positive emotion of hetero-induced pride is triggered. Indeed, something quite close to this form of reasoning seems to happen fairly often. In the social psychology of sports, there are two well established phenomena: basking in reflected glory (BIRGing) and cutting off reflected failure (CORFing). Some empirical research has shown that people are much more likely to wear team paraphernalia and talk about the sports team they support in “we” terms (“we won”) the day after a victory than the day after a defeat. After a defeat, the tendency is to speak about “they” instead: “the team played badly” (cf. Cialdini et al., 1976; Snyder et al., 1986; Bizman and Yinon, 2002). This might explain why hetero-induced pride is so frequent.

However, not everyone agrees, and for good reasons. What if the self-conscious emotion that is induced by others’ behavior is negative? The phenomenon of CORFing seems to show that people tend to dissociate from others in this case.^10^ But is this always so? What if, e.g., the subject feels shame for what others have done? If something like hetero-induced shame exists and if its explanation has to appeal to the notion of group identification, this would suggest that the social self can – but does not have to – be the result of a psychological process driven by the desire to establish a positive social identity. But if that is the case, then the desire to “maintain or enhance self-esteem” or even the struggle “for a positive social identity” would be extrinsic to group identification, i.e., it would be an element that could facilitate or, as it were, grease group identification, but which is not necessary for this process to occur. To test this idea, let us now turn our attention to hetero-induced shame.

## Being Ashamed of Your Daughter

As explained above, shame can be described as a self-conscious emotion involving a negative self-assessment. One is typically ashamed of one’s defects, failures, or mistakes. But in the same way that one can feel proud of others, one can also feel ashamed of them, or so we will argue in this Section. Imagine it turned out that your physicist daughter had fabricated the data published in some of her more important papers, invalidating all her contributions to science, and starting a scandal. In this case, it is plausible to think that you would feel ashamed of her. Now, some of the objections discussed for pride might apply to hetero-induced shame as well: perhaps this is after all standard shame (shame of your bad parenting skills, of your inability to instill good values in her), perhaps this emotion is of an infectious type, i.e., elicited by emotional contagion, or perhaps this is fictional shame. These objections have been dismissed in the previous Section and the same responses given for pride seem to apply equally well to shame. These arguments will not be rehearsed here, but to recap some of their main points: standard shame does not capture the role that the other plays in the intentional structure of the emotion. Emotional contagion requires the other to feel an infectious emotion of the same kind, but this is not needed for hetero-induced shame. Fictional shame presupposes some cognitive processes that do not need to be in place in the case of hetero-induced shame.^11^

In addition to those challenges, there are a couple of other objections that are specific to the case of shame, partially because, as mentioned above, the language of shame-related emotions is more nuanced than the language of pride. These are: (a) such cases are examples of indignation at shamelessness and (b) they are examples of embarrassment, not shame. Let us look at each one in turn.

Consider the idea that so-called hetero-induced shame is actually indignation at shamelessness. The gist of this objection would be that many cases of hetero-induced shame that, as mentioned above, some languages refer to with words such as ‘Fremdscham’ or ‘vergüenza ajena,’ actually do not refer to a shame reaction, but rather to an indignation-like response at a display of shamelessness. If indignation tracks offense and injustice, and therefore responds to a violation of what one could call the code of guilt (cf. Nussbaum, 2006, p. 99–101), the emotion under examination here would be an analog directed at violations of the code of shame instead. When someone behaves disgracefully, you would experience an aversive reaction that implies a condemnation of that action. Some examples might make this explanation plausible. Imagine that one evening, while browsing TV channels, you stumble upon a particularly outrageous reality show and, unable to bear watching the behavior of the participants, you swiftly change the channel again. It seems that the element of condemnation is here very prominent and makes this idea attractive. However, such an explanation doesn’t seem to capture the other scenario introduced above: there is a difference between finding out that the fraudster is just some random person (which can grant indignation) and finding out it is *your daughter*. Just as admiration, which is exclusively externally directed, is not equivalent to hetero-induced pride, indignation is not equivalent to hetero-induced shame: the crucial idea here is that hetero-induced shame retains a feeling of your own involvement with the shameful subject and can make you wish to hide from others, even though you are not the one who behaved shamefully. And all this is *not* preserved in mere indignation.

But then, is hetero-induced shame perhaps better described as embarrassment? This objection would run as follows: there is another emotion that is closely related to shame, but with some peculiarities that help accommodate the examples just presented, namely embarrassment. Embarrassment is another negative self-conscious emotion, but it is milder and more contextual. While shame has a deeper and longer-lasting impact in the subject’s self-esteem, because it touches on her qualities, embarrassment is typically a feeling of being out of place or awkward in a social situation, and it generally disappears without impacting the subject’s sense of self after the situation is over (cf. Nussbaum, 2006, p. 204–206). While in shame one experiences oneself as being at fault or inferior, in embarrassment one merely experiences oneself as being socially out of place. Therefore embarrassment, as opposed to shame, which can be felt in solitude, always requires an audience: someone has to be directly witnessing the situation. And once the actual social exposure is over, embarrassment usually wanes quickly without leaving a sense of degradation. Due to the centrality of contextual elements in embarrassment, one might think that it is easier for situations to evolve in ways that potentially leave one out of place through no fault of one’s own. Imagine that, when you first introduce your new romantic partner to your family, one of your siblings starts telling stupid and inappropriate jokes. This might put you in a socially awkward position, but you don’t have to construe it as reflecting on your character. So, the objection would go, your feelings in this case are better described as embarrassment rather than shame.

Now, the first response to this would be that, as Miller (1985, p. 28) claims, emotion terms are not so effortlessly applied to experiences as concepts such as “chair” or “table” are applied to objects, and thus it is problematic to assume that emotion terms designate clear-cut areas of experience. Boundaries are blurry, and there are often wide areas of confusion and overlap between closely related emotions. This is the case of shame and embarrassment, which belong to the same emotional family, they resemble each other, and embarrassment can sometimes slide into shame, if the socially awkward situation is perceived as revealing some flaw of yours. The above example might plausibly look like embarrassment (although this situation could elicit hetero-induced shame too, if you group-identify with your sibling), but it is much harder to see how an explanation in such terms can do justice to our introducing example. Imagine that you read about your daughter’s fraud case in the press while alone in your living-room. This would plausibly give rise to a shame-like emotion. What would justify classifying it as embarrassment instead? There are no witnesses that make this situation socially awkward, the emotion cannot be avoided by removing yourself from the social context, and the evaluation involved surely would go beyond a feeling of being out of place. We therefore think that there are paradigmatic cases of hetero-induced shame that cannot be explained in terms of embarrassment.

At this point, it seems one can conclude that hetero-induced shame is a genuine phenomenon that cannot be explained away or reduced to a different phenomenon. It has a specificity vis-à-vis regular shame, but it still shares the core of its intentional structure, i.e., it is directed at the emoting subject. The difference can be argued to be that hetero-induced shame, just like hetero-induced pride, relies on a mechanism of group identification. You feel ashamed of your daughter because you perceive her as a member of a group that you also belong to (your family) and this is relevant for your social identity. You are ashamed of yourself qua member of that group because she threw a negative light on it. Conversely, and perhaps even more poignantly, the daughter could be ashamed of her father/mother because of his/her alcoholism.^12^

## Group Identification in the Light of Hetero-Induced Self-Conscious Emotions

The last *tessera* of the mosaic being at its place, one can now get back to the question regarding the nature of group identification. Is it the desire to create or maintain a positive social identity one of the keys to understanding group identification, as some have argued? Although the positive quality of hetero-induced pride could be taken to speak in favor of this idea, the fact that hetero-induced shame is an emotion that involves a resolutely negative evaluation of the shameful subject seems to invalidate the hypothesis. And the argument seems to be the following: since shame responds to features and traits that are perceived to be negative, the hypothesis at stake would predict that the subject does not desire to be associated with them. And yet, there are genuine cases of hetero-induced shame that can’t be avoided by the abovementioned strategy of CORFing. Now, if the explanation of these cases, at least according to our suggestion, requires appeal to the process of group identification, then the conclusion to infer is that group identification does not draw upon the desire to establish a positive social identity – at least not necessarily.

This conclusion, however, could be resisted based on the following line of reasoning. It might well be that hetero-induced shame presupposes group identification, but this does not militate against the hypothesis that group identification is triggered by a desire to achieve a positive self-identity. Once a social self has been established, one can come to feel negative self-conscious emotions induced by other group members, but this has no bearing on the mechanisms that prompted group identification in the very first place. This objection seems very plausible if one thinks of the paradigmatic cases of feeling ashamed of one’s family members, where the subject arguably has a social self *before* any hetero-induced self-conscious emotion comes into play. If that is on the right track, it might still be the case that the motivation underlying group identification is to strive for positive self-identity.

However, cases like this, it seems, delimit only a mere sub-class of hetero-induced self-conscious emotions. More precisely, there are many cases of hetero-induced shame or pride where the emoting subject does not have a previously established social self and where, hence, group identification seems to occur in concomitance with the emotions without presupposing any form of previous (*subjective*) group membership (on this, cf. Montes Sánchez and Salice, forthcoming). As an illustration, consider these two examples:

– Someone who is not nationalist, nor at all interested in football, gets carried away by the frenzy surrounding the finals of the World Cup, where her country’s national team is playing, and ends up watching the match. As it happens, the team wins the competition and she feels proud. Neither her nationality, nor football, had ever been a salient part of her social identity in any situation, and yet in this particular moment she feels hetero-induced pride.
– As you walk down the street one day, you see a beggar sitting on the sidewalk a few meters ahead. Suddenly, the man who was walking just in front of you spits on the beggar. Upon seeing this, you feel ashamed. The man who spit on the beggar is a complete stranger, someone you have never seen before, and yet in this situation you feel ashamed of him.^13^

These are cases where, arguably, the social self at stake in the emotions was absent before the situation took place, and where some feature of the situation seems to be triggering group identification. In the first case, it is plausible to argue that the person watching the match group-identifies because she desires to associate to the positive qualities of the agents. But this pattern of explanation cannot hold with respect to the second example. Indeed, in that situation one might intensely desire to reject the association, though one finds oneself unable to do that, as hetero-induced shame evidences. In other words, if one strongly wishes to dissociate from the man who spits on the beggar, then how come one is group-identifying with him? In this case, it does seem fairly implausible to argue that group identification is motivated by the desire to acquire a positive social identity and this provides additional evidence in favor of the suggestion that group identification does not necessarily rest on this desire. However, this being a mainly negative result, does it also have a positive twist? The remaining of this Section is devoted to mapping the terrain of three possible options.

To begin with, one could conjecture that – even if this particular desire or, more precisely, a desire with this particular content (to establish and preserve positive social identity) is not necessary for the subject to group-identify – this process still has to be triggered by a desire (the exact content of which would still have to be specified). Were this correct, every occurrence of group identification would have to be described as an intentional activity – “intentional” understood in a very strict sense, according to which this activity can only be caused by a conative attitude. To the best of our knowledge, such a strong claim does not find advocators in the literature. And maybe there are good reasons for that. To come back to our previous example, it seems possible to imagine scenarios in which one feels ashamed for the actions of complete strangers – but if there is any desire involved in this sort of case, it would be a desire to be *dissociated* from the shameful agent. And it is hard to make sense of how such a desire would elicit group identification.

Recently, a nuanced position has been suggested by Tuomela (2013) in the context of decision-making theory. According to Tuomela (2013, p. 195), in certain cases, but also *only* in certain cases, group identification (or, in his parlance, the adoption of a we-mode) is intentional. Although it has been pointed out that this view is not without tension (Townsend, 2014, Petersson, unpublished manuscript), it appears to fit the results of our analysis: in fact, one could claim that relevant desires on the side of the group-identifier do play a causal role for hetero-induced pride, whereas no such desires can be ascertained in the case of hetero-induced shame. This approach, however, would still have to explain *why* in certain circumstances group identification is an intentional process and why in other cases this description does not hold. Note, however, that on this view group identification is not necessarily intentional.

This leads to another possible solution of the problem – one, that denies group identification *ever* being intentional and that rather takes it to be a brute fact, which cannot be explained further by recurring to conative attitudes of the subject (cf. Bacharach, 2006, p. 86). On this view, the best that a theory of group identification can achieve is to identify the psychological factors that make group identification more likely. And these would be the factors encompassing those briefly illustrated in Section 2: social categorization, second person perspective taking, common fate, etc. These psychological conditions *may* trigger group identification, but none of them is able to *exact* this process. Accordingly, group identification would be an arational (and yet not irrational) process.

A systematic assessment of these accounts about group identification would require consideration of several other issues, touching upon theories as disparate as the theory of intentions, of self and of agency, among other. This is a topic for another paper, but it might be worth emphasizing that the account of a hetero-induced form of self-conscious emotions propounded in this article appears to be fully compatible with the last two accounts presented.

## Conclusion

In this paper we have argued, on the one hand, that two important self-conscious emotions, shame and pride, can be social in a specific way, and on the other hand, that these phenomena contribute to shed some light on the mechanisms underlying group identification.

*First*, it has been claimed that shame and pride can be hetero-induced through a process of group identification. This shows that the evaluation of one’s social self can be impacted by the evaluations deserved by other members of the group with which one identifies. In other words, one’s self-evaluation does not exclusively depend on one’s own actions and features; it is interlinked with those of others – insofar as they are considered members of the same group to which the subject ascribes herself. From the point of view of moral psychology, if one thinks about self-evaluation in moral terms, this might seem problematic. Why should the actions of others have an impact on my moral self-evaluation, if I’m not responsible for them? Answering this question in detail would require a discussion of collective responsibility and a proper investigation into the conditions of appropriateness of hetero-induced shame and pride, all of which greatly exceeds the scope of this paper. Let us just say that we believe hetero-induced emotions *can* be appropriate in some cases, and this should be taken into account by moral psychology: our moral self is not self-encapsulated, as some prominent discussions of the moral role of shame and guilt seem to assume (cf. Tangney and Dearing, 2004).

*Second*, it has been shown that the possibility of feeling hetero-induced shame in certain situations casts serious doubts on the idea that group identification is triggered by the desire to maintain a positive social identity. Such desire can contribute to make group identification quicker, easier, or more likely, but this is by no means necessary for it to happen. We have suggested some alternative pathways that might lead to group identification, but a careful investigation of them will have to remain the subject of further research.

## Author Contributions

All authors listed, have made substantial, direct and intellectual contribution to the work, and approved it for publication.

## Conflict of Interest Statement

The authors declare that the research was conducted in the absence of any commercial or financial relationships that could be construed as a potential conflict of interest.

## Footnotes

1 Pride and shame are not the only self-conscious emotions, but they are the only two we focus on here. Other examples include embarrassment, guilt, humiliation, and envy. Not all of them would clearly qualify as “emotions of self-assessment" in Taylor’s (1871) sense and we leave it to further investigations whether they can be hetero-induced.

2 Not everybody agrees on this, though. Tracy and Robins (2007), for example, claim that pride (as opposed to shame) is subdivided in two clearly distinct types, “authentic pride” and “hubristic pride,” involving very different action tendencies and functions, whose opposite emotions are, respectively, guilt and shame. What they call “authentic pride” would not be intentionally parallel to shame, but to guilt. Other authors suggest a different way in which shame and guilt might not be parallel: namely, they take one of the self-conscious emotions to be more basic than others, and to form the ground from which others derive. Tomkins (1995, p. 397–410) could be read as suggesting that pride, as a positively valenced affect, is prior to shame, since he believes that shame arises from the thwarting of positive affect. Sartre (1969, p. 290–91), on the other hand, argues that shame is primary, and all other self-conscious emotions, including notably pride, are transformations of it. This is no place to make a detailed evaluation of these views, whose theoretical presuppositions are too far from the subject of this paper. Suffice it to say that neither our analysis of the intentional structure of pride and shame, nor empirical findings in developmental psychology (cf. Reddy, 2008, p. 120–49) seem to clearly support the primacy of one of these emotions over the other.

3 Some authors deny that emotions have propositional contents, while still holding that they involve evaluation or appraisal (cf. Prinz, 2004).

4 Hume does not use the word “shame,” but rather the word “humility.” He takes pride and humility to be the two opposite emotions, of positive and negative self-assessment, respectively. From his discussion it is clear that he uses both terms very broadly, and that shame not only falls within the scope of what he calls humility, but also is the most natural current term for many of his examples of humility. Therefore, in what follows, we take his claims about humility to apply to shame.

5 We use this qualification to distinguish cases like those under considerations in this paper from ‘collective’ pride or shame, i.e., from emotions that, allegedly, are felt by a group and, hence, are collective (cf. Sullivan, 2014). If considered from a linguistic point of view, collective emotions would be expressed by sentences of the form ‘*we* feel emotion ϕ;’ hetero-induced emotions, by contrast, come in the I-form ‘I feel ϕ [pride or shame] of myself [because of you].’ Some social psychologists also refer to ‘hetero-induced’ emotions as ‘group-based’ (as opposed to “collective,” cf. Lickel et al., 2011) – we employ the former expression here because, as pinpointed below, although we contend all hetero-induced emotions require (group-)identification with the other, we also argue that not all of them are based on an actually existing group.

6 As Kristjánsson (2010, p. 83) remarks, it is a well-documented fact that the language of positive emotions is far less nuanced than the language of negative ones. As he says, “there are, it seems, fifty ways to feel bad for every one to feel good!”.

7 These remarks merely highlight one key aspect that distinguishes paradigmatic cases of emotions elicited by fiction from hetero-induced ones. They are not supposed to specify our position in the debate on the nature of emotional responses to fiction.

8 Despite our obvious agreement with this psychological account, it seems to leave some important questions about group-based self-conscious emotions unasked and undertheorized, mainly because it doesn’t take into sufficient account the peculiarity of self-conscious emotions we address here (however, cf. also Lickel et al., 2005, 2007). Taking it into account, and asking how exactly a self-conscious emotion can become group-based, or hetero-induced, based on a process of group identification, puts on the table issues of attributability and responsibility that pave the way to exploring the adequacy criteria of group-based self-conscious emotions and their consequences for moral psychology. We will not explore the latter two topics in this paper, but these are clearly related to the first one, i.e., the issue about group identification. As we emphasize below, group identification does not appear to require the actual existence of a group (cf. also Montes Sánchez and Salice, forthcoming). But if that is the case, then what are the conditions of adequacy for hetero-induced self-conscious emotions? When, in other words, can these emotive responses be said to be ‘appropriate’? One suggestion could be that at least *one* of these conditions is related to group membership – for the subject to elicit an adequate emotion of this kind, it is not yet sufficient that the subject group-identifies, for objective group-membership must also be in place. Yet, this certainly represents only one within a whole set of further conditions, which we are not in a position to explore here. This, again, has an impact on questions related to moral psychology: why should the virtues and vices of other people have an impact on the individual’s self-evaluation? Isn’t it healthier and more mature to be autonomous in this respect? Problematizing the origin of hetero-induced shame and pride as this paper is aiming at doing, is, we believe, a first necessary step to start clarifying questions like these. (We thank one anonymous reviewer for pushing us on these points.)

9 But suppose that someone who, *prima facie*, is not member of your group has reached the admirable result. Is it yet possible for you to feel proud of her? Answering this question presupposes a clarification of the notion of group membership, which goes beyond the purposes of this paper. However, in previous work (Montes Sánchez and Salice, forthcoming) we have argued that the process of group identification, although it can establish group membership, does not necessitate group membership, meaning that it seems possible for a subject to identify with a group without him or her yet being member of that group (or even without there being a group in the very first place). If that is the case, given certain psychological preconditions, it might be possible for a subject to feel pride for the actions of individuals that, although do not actually belong to the same group as the subject, are nevertheless considered by the subject to be members of her group.

10 We owe the ideas about CORFing and BIRGing to Gerhard Thonhauser and to his work on sport fandom.

11 An interesting account of hetero-induced shame along fictional lines is developed by Kutz (we are thankful to Olle Blomberg who has pointed us to Kutz’s theory). According to Kutz, when individual shame is induced by others, this emotion has to be explained by a combination of “basic shame and identification,” where identification is understood as an “imaginative projection.” The idea is that, in these cases, I feel ashamed “not for the other (…), but for myself as someone in whom the same shameful characteristics are likely to be expected by association” with whatever category the other belongs to (cf. Kutz, 2000, p. 44). It seems that, although this can happen, it does not have to. To use Kutz’s own example, it could be that, “while in a restaurant in a foreign country, I hear another tourist loudly and abrasively, I hide my guidebook in shame,” but this happens not, as Kutz argues, because I feel I could instantiate the same shameful characteristics of the category “tourist,” for I could be adamantly certain that I would never act in the same way. Rather, and as it will be suggested below, the shameful actions of the other gain relevance for my social self, because the other is perceived as being a member of the same group to which I belong. Insofar as the other performs actions that are shameful for the group I identify with, they are shameful for me, too.

12 Further support to these ideas comes from psychopathology and, in particular, from two independent studies conducted about schizophrenia. The first study highlights that the self-disorders that constitute the Gestalt of schizophrenia (and especially: ontological dissimilarity, loss of common sense, transitivism, quasi solipsistic experiences; on these cf. Parnas et al., 2005) impair the patients’ ability to group-identify (cf. Salice and Henriksen, 2015). The second study illustrates that patients with schizophrenia have difficulties in feeling hetero-induced shame (or *Fremdscham* tout court, given that the study was conducted in Germany, cf. Krach et al., 2010). Jointly taken, these two studies seem to confirm the link between hetero-induced shame and group identification.

13 This example is a variation of a scenario first portrayed by Henning Nörenberg.
